# Reorganization of the nuclear architecture in the *Drosophila melanogaster* Lamin B mutant lacking the CaaX box

**DOI:** 10.1080/19491034.2020.1819704

**Published:** 2020-09-22

**Authors:** Semen M. Bondarenko, Igor V. Sharakhov

**Affiliations:** a Department of Entomology, Virginia Polytechnic Institute and State University, Blacksburg, Virginia, USA; b Department of Cytology and Genetics, Tomsk State University, Tomsk, Russian Federation

**Keywords:** Nuclear lamina, Lamin B, *Dm_0_*, B-type lamin, *Lam^a25^* mutant, *Drosophila*, chromatin, nuclear envelope, proventriculus nuclei, salivary gland nuclei, confocal microscopy

## Abstract

Lamins interact with the nuclear membrane and chromatin but the precise players and mechanisms of these interactions are unknown. Here, we tested whether the removal of the CaaX motif from Lamin B disrupts its attachment to the nuclear membrane and affects chromatin distribution. We used *Drosophila melanogaster Lam^A25^* homozygous mutants that lack the CaaX box. We found that the mutant Lamin B was not confined to the nuclear periphery but was distributed throughout the nuclear interior, colocalizing with chromosomes in salivary gland and proventriculus. The peripheral position of Lamin C, nuclear pore complex (NPC), heterochromatin protein 1a (HP1a), H3K9me2- and H3K27me3-associated chromatin remained intact. The fluorescence intensity of the DAPI-stained peripheral chromatin significantly decreased and that of the central chromatin significantly increased in the proventriculus nuclei of the mutantflies compared to wild-type. However, the mutation had little effect on chromatin radial distribution inside highly polytenized salivary gland nuclei.

## Introduction

The nuclear lamina is an important structural component of the nuclear envelope in the metazoan cell nucleus. It consists of a large number of proteins, including lamins – the major building blocks of the nuclear lamina – and associated transmembrane and chromatin-binding proteins [1–3]. Lamins are type V intermediate filaments that cover the inner surface of the nuclear membrane in a majority of animal tissues. Lamins form helical dimers that interact with each other in a head-to-tail orientation assembling into a protein meshwork. Current models of nuclear organization suggest that nuclear lamina is important for the maintenance and functioning of the 3D chromosome architecture by interacting with both the nuclear membrane and chromatin [4–8]. However, the precise players and mechanisms of these interactions are unknown. There are two known types of lamin proteins: A- and B-type. Both lamin types contain an ⍺-helical rod domain between the N-terminal head domain and the C-terminal tail domain. The main structural difference between these lamin types is the presence (B-type) or absence (A-type) of the CaaX (C, cysteine; a, an aliphatic residue; X, any residue) motif at the end of the C-terminal tail domain. The nuclear lamina in mammalian species includes four isoforms of lamin. Two A-type lamins (A and C isoforms) are encoded by one LMNA gene, whereas the B1 and B2 isoforms are encoded by different genes – LMNB1 and LMNB2, respectively [5]. All currently known invertebrate species have one B-type lamin gene [2], except for Tunicates, which have two B-type lamin genes (L1 and L2), where L1 encodes for two protein variants, L1α and L1β [9]. The fruit fly, *Drosophila melanogaster*, has two types of lamin encoded by different genes. The type A lamin is encoded by the gene *Lamin C* (or *LamC*, also known as *pG-IF*, FlyBase ID: FBgn0010397), and the type B lamin is encoded by the gene *Lamin* (or *Lam*, also known as *lamin B, lamin Dm_0_, Dm_0_, lamin Dm0, Dm0*, FlyBase ID: FBgn0002525) [2,10]. Similar to mammals, B-type lamin in *Drosophila* is expressed throughout development in all cell types, whereas A-type lamin is expressed starting during late stage 12 in fly embryos [11]. The availability of lamin mutants [12] makes *D. melanogaster* a convenient model system to study the roles of the protein domains in the interaction of lamins with the nuclear membrane, nuclear proteins, and chromatin.

Biochemical investigation of lamins is a challenging task due to the non-globular organization of these proteins [5]. Thus, the precise functions of either the entire lamin or its domains are poorly understood [5,13]. Also, most biochemical studies of lamins are based on *in vitro* experiments that pose limitations on the interpretation of results. Multiple works report that the CaaX motif at the end of the C-terminal tail domain is responsible for tethering Lamin B to the nuclear envelope [3,4,10,14]. However, the presence of the CaaX box is essential but not sufficient for the attachment of Lamin B to the nuclear membrane [15]. Therefore, other lamina-associated proteins may also be important. Even more unclear are the mechanisms of interaction between Lamin B and chromatin. If direct interaction is possible, there is also the challenge of determining the Lamin B domain responsible for this interaction. It has been reported that the ⍺-helical rod domain mediates the association of the protein with chromatin [16]. The C-terminal tail domain, including the CaaX box, may also be responsible for this function [15,17]. In addition, the CaaX motif plays a crucial role in the process of lamina filament assembly providing head-to-tail interaction between linearly ordered lamin dimers in the eventual composition of the *in vivo* polymer [5].

A recent study has shown that elimination of both types of lamin from the nuclei in a *Drosophila* cell line leads to translocation of chromatin from the nuclear periphery and transcriptional derepression of lamina-associated genes, which directly demonstrates the importance of lamins for tethering chromatin to the nuclear envelope [18]. However, this approach does not clarify which lamin domains are responsible for the interaction with the nuclear membrane and chromatin in fruit fly. Moreover, it is unknown whether the effects of lamins on chromatin distribution are similar in living organisms or whether these effects are cell-type specific. Complete removal of lamins is impossible in *D. melanogaster* flies as it will cause lethality at the early embryogenesis stage [12]. Lamin mutants, however, offer important advantages for such studies. First, the presence of a mutated (partially dysfunctional) lamin protein in the organism ensures the survival of mutant homozygotes and confers loss of function of the protein of interest at the same time. Second, frameshift mutations located in different regions of the gene allow investigations of the roles of different lamin regions and domains in interactions with the nuclear membrane, other nuclear proteins, and chromatin. It has been shown that deletion of the A-type lamin gene *LamC* causes lethality at the prepupal stage [19]. Mutations in the B-type lamin gene *Lam* (or *Dm_0_*) generated by P element insertions can result in dorsal-ventral polarity and tracheal branching embryonic defects as well as developmental retardation, reduced viability, sterility, and impaired locomotion in flies [12,20,21]. A homozygous mutation in Lamin B obtained by X-ray irradiation, *Lam^A25^*, which removes the CaaX, causes defects in photoreceptor nuclear migration in the *Drosophila* eye [12]. In addition, the *Lam^A25^* protein dysfunction can lead to heterochromatin decondensation, DNA damage, and neuronal death in *Drosophila* adults [22]. A recent study found widespread accumulation of polyadenylated RNA in the brains of *Lam^A25^* homozygotes compared to controls, suggesting that Lamin B dysfunction causes aberrant RNA trafficking and localization [23].

In this study, we examined the role of the CaaX box in the positioning of Lamin B and chromatin inside the polytene nuclei of the salivary gland and the proventriculus (the end part of the larval foregut [24]) in *D. melanogaster* larva using high-resolution imaging. We asked four speciﬁc questions: (i) Does the lack of the CaaX motif in the *Lam^A25^* mutants affect the interaction of Lamin B with the nuclear envelope or chromatin? (ii) Does the *Lam^A25^* lamin mutation cause spatial repositioning of the chromatin? (iii) Does the *Lam^A25^* lamin mutation affect localization of other peripheral proteins or modified histones? (iv) Are any effects of lamin dysfunction on nuclear architecture cell type-specific? We demonstrated that the CaaX box is necessary to tether Lamin B to the nuclear envelope and that the Lamin B protein lacking the CaaX motif colocalizes with polytene chromosomes in both tissues. Our data also indicate that mutant lamin does not affect the localization of other peripheral proteins but causes a significant shift in chromatin distribution from the periphery to the nuclear center in the proventriculus, but not in the salivary gland.

## Materials and methods

### *Fly stocks and the crossing scheme to obtain* Lam^A25^ *homozygotes*

**Figure 1.**
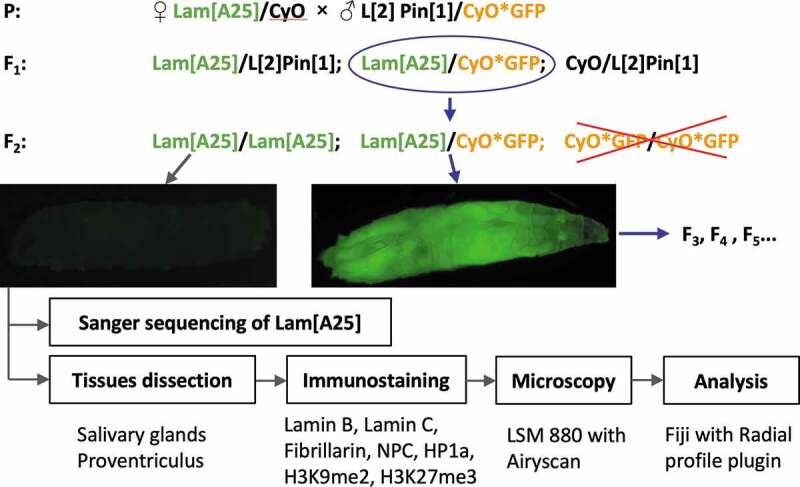
Crossing scheme and analysis workflow to characterize homozygous *Lam^A25^ D. melanogaster* mutants.

*D. melanogaster* lines were obtained from the Bloomington Stock Center (BSC). The original lamin-mutant line had genotype *Lam^A25^pr^1^/CyO; st^1^* (BSC, #25092). For easier identification of homozygous mutant larvae, the original *Lam^A25^ pr^1^/CyO; st^1^* line was crossed with flies expressing green fluorescent protein (GFP) in the balancer chromosome – w*; L^1^Pin^2^/CyO,P{w^+mC^ = GAL4 – Kr.C}DC3,P{w^+mC^ = UAS–GFP.S65T}DC7 (BSC, #5194) (Figure 1). The experimental homozygous lamin-mutant flies had the genotype w*; Lam^A25^pr^1^; st^1^. Canton-S (BSC, #64349) flies were used as the wild-type (*wt*) control. Experimental and control groups were maintained at 26°C on the ‘German Food’ Sick Fly Formulation (Genesee Scientific, San Diego, CA, USA) according to standard protocols described in Flagg, 1988 [25].

### *Sequencing and localization of the* Lam^A25^ *frameshift mutation*

Genomic DNA was extracted from ten w*; Lam^A25^pr^1^; st^1^ homozygous larvae using the DNeasy Blood & Tissue Kit (#69506, Qiagen, Hilden, Germany). The 300–500-bp-long fragments were PCR-amplified with 12 pairs of primers (**Table S1**) using 2× ImmoMix reaction-mix (#25020, Bioline USA Inc., Memphis, TN, USA). PCR products were purified using a QIAquick PCR Purification Kit (#28104, Qiagen, Hilden, Germany) and prepared for sequencing in accordance with the Sanger sequencing protocol of the Virginia Tech Genomic Sequencing Center (VT GSC, Blacksburg, VA, USA). Sanger sequencing was performed on a 3730 DNA Analyzer (Applied Biosystems, Foster City, CA, USA) and analyzed using UGENE software (Unipro, Novosibirsk, Russia) [26]. The assembled sequence of the *Lam^A25^* allele is available under GenBank accession number MN653920.

### Whole-mount immunostaining

Salivary glands and proventriculi were dissected in 1× Phosphate-buffered saline (PBS) from *Lam^A25^* mutants and *wt* 3^rd^ instar larvae. The dissected tissues were fixed in 4% paraformaldehyde dissolved in PBST solution (0.3% Tween 20 in 1 × PBS) for 20 minutes and washed in pure PBST for 5 minutes three times. The fixed tissues were incubated in PBSTr solution (2% Triton X-100 in 1× PBS) for 20 minutes and washed in PBST three times for 5 minutes each. Tissues were incubated in blocking solution (0.2% bovine serum albumin, 0.1% Normal Goat Serum in PBST) for 2 hours and then in blocking solution with anti-Lamin B (Dm0) primary mouse antibody ADL67.10 or ADL84.12 (DSHB, Iowa City, IA, USA), anti-fibrillarin primary rabbit antibody ab5821 (Abcam, San Francisco, CA, USA), anti-Lamin C primary mouse antibody LC28.26 (DSHB, Iowa City, IA, USA), anti-heterochromatin protein 1a (anti-HP1a) mouse antibody C1A9 (DSHB, Iowa City, IA, USA), anti-H3K9me2 rabbit antibody A-4035-025 (Epigentek, NY, USA), anti-nuclear pore complex protein (anti-NPC) mouse antibody 1515-NPC (PhosphoSolutions, CO, USA) and anti-H3K27me3 mouse antibody ab6002 (Abcam, San Francisco, CA, USA) diluted 1:200 at 4°C overnight. The tissues were washed in PBST for 5 minutes three times and incubated in blocking solution with secondary goat anti-mouse IgG H&L FITC antibody ab6785 and goat anti-rabbit IgG H&L Alexa Fluor® 594 antibody ab150080 (Abcam, San Francisco, CA, USA) diluted 1:500 and washed again in PBST for 5 minutes three times. The samples were stained by incubation in Prolong Gold antifade reagent with 4′,6-diamidino-2-phenylindole (DAPI) (Invitrogen, Eugene, OR, USA).

### Confocal microscopy

Visualization of the whole-mount preparations was performed under a 63× water-immersion objective of a Zeiss LSM 880 confocal laser scanning microscope with the Airyscan module (Carl Zeiss AG, Oberkochen, Germany), which allows one to achieve a resolution of 140 nm laterally and 400 nm axially. Z-stacks of optical sections with 1 µm intervals were acquired and visually evaluated with Zen Blue Edition 2.6 (Carl Zeiss AG, Oberkochen, Germany).

### Measurement of the radial chromatin distribution and statistical analyses

Quantification of the fluorescence intensity along the nuclear radius was used to measure the radial distribution of DAPI-stained chromatin inside the nucleus. Quantification of fluorescence intensity distribution from the center to the periphery of each nucleus was processed using Fiji software [27] (a branch of ImageJ [27,28]) with a ‘Radial profile’ plugin [29]. Images of the nuclei were collected from three independent immunostaining experiments for each tissue. In total, we measured 98 proventriculus nuclei from 9 mutant individuals, 78 salivary gland nuclei from 7 mutant individuals (**Figure S1**), 103 proventriculus nuclei from 10 *wt* individuals, and 86 salivary gland nuclei from 12 *wt* individuals (**Figure S2**). The normalized integrated fluorescence intensity was measured for the equatorial focal plane of nuclei stained by DAPI. The intensity at any given distance from the center of the nucleus represents the sum of the pixel values around a circle [29]. The radial coordinates for each nucleus in pixels were translated into relative coordinates in percentages (0–100%) and were aggregated in each group by distributing relative coordinates of each nucleus within the axes divided by 5% intervals. The mean and standard error values were calculated in each interval within each experimental group (**Figure S3**). The intensity levels in mutant and *wt* nuclei for each radial interval were compared using the statistical Mann–Whitney U-test. The value of Δ (Figure 4, **Figure S3**) was aimed to estimate the integral difference (%) of the DAPI signal relative intensity of the *Lam^A25^* group of nuclei in comparison with the *wt* group within a specific local interval of radial positions and was calculated by the following formula: Δ = (1 – (Σ_x∊L_I*_wt_*[x]/Σ_x∊L_I*_Lam_*[x])) × 100%, where I_G_[x] ∊ [0, 1] denotes the relative integral intensity of the DAPI signal on the radial position x ∊ {5, 10, … , 100} for group G ∊ {*wt, Lam^A25^*} and L specifies the range of relative radial positions on which the difference is calculated.

## Results

### *Authentication of the* Lam^A25^ *mutation by sequencing*

In this study, we used a homozygous partial loss-of-function allele, *Lam^A25^*, to genetically induce Lamin B dysfunction in *Drosophila*. The selection of homozygous larvae is described in the materials and methods section and Figure 1. GFP-negative larvae were able to develop into adults in agreement with previous studies that demonstrated the survival of the homozygous *Lam^A25^pr^1^* adults [12,30]. We obtained adults from *Lam^A25^pr^1^* homozygous larvae in three independent vials by growing them for two weeks. The *Lam^A25^pr^1^* homozygous adult females were allowed to mate with *Lam^A25^pr^1^* males and lay eggs; these eggs did not hatch. Thus, we demonstrated that the homozygous *Lam^A25^* mutation causes sterility. Because we planned to use cell types with polytene chromosomes for our study, we wanted to check if the banding pattern of the polytene chromosomes was affected by the *Lam^A25^* mutation. Our analysis of squashed salivary gland preparations obtained from homozygous *Lam^A25^* larvae did not detect any obvious anomalies in the banding pattern of polytene chromosomes (**Figure S4**) compared to a standard cytogenetic map [31].

**Figure 2.**
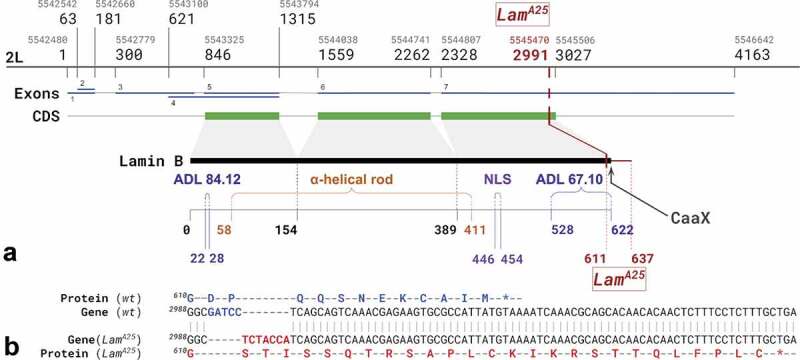
The structure of the *Lam* gene and *Lam^A25^* allele in *D. melanogaster*. (a) The location of the *Lam^A25^* frameshift mutation in the *Lam* gene. NLS – nuclear localization signal; ADL67.10 and ADL84.12 – epitopes recognized by the Lamin B antibodies used in this study; CDS – coding sequence. Grey numbers indicate genomic coordinates on chromosome 2L in FlyBase, v. 6.29. Black numbers correspond to nucleotides of the *Lam* gene sequence. The first genomic coordinate and gene nucleotide number as well as the first and last amino acid number of the *Lam^A25^* substitution are shown in red. The tail domain is from 411 to 622 amino acids. (b) The fragment of wild-type (*wt*) Lamin B nucleotide sequence (from ^2988^C to ^3008^C) is shown in comparison with the corresponding sequence of the *Lam^A25^* mutant. The sequences involved in the frameshift are shown in blue and red.

The *Lam^A25^* mutation was characterized in Patterson et al. (2004) as a substitution of (^1831^GATCC) to (^1831^TCTACCA) leading to a frameshift in the protein sequence after ^610^Gly. To verify this mutation in the *D. melanogaster* BSC line (#25092), we sequenced overlapping fragments of the entire *Lam* gene using 12 pairs of primers (**Table S1**). We confirmed the presence of the *Lam^A25^* mutation in the experimental flies. According to our sequencing results (Figure 2), substitution of the (GATCC) fragment to the (TCTACCA) fragment occurs after the ^2990/1830^C nucleotide of the gene/coding sequence (CDS) (FlyBase v. 6.29 gene coordinate 2L:5545469). The identified *Lam^A25^* mutation led to a frameshift after ^610^Gly resulting in 27 new amino acids in the Lamin B protein sequence in agreement with Patterson et al. (2004). The epitope for recognition of Lamin B by the ADL84.12 antibody encompasses amino acids 22–28 [32]. The majority of the epitope for recognition of Lamin B by the ADL67.10 antibody is preserved in the protein. The epitope for ADL67.10 encompasses amino acids 528 and 622 [32], while the mutation affects the protein starting with amino acid 611 (Figure 2). Therefore, it is possible to use the same antibodies for localization of both *wt* and mutant lamin.

### Localization of the mutant Lamin B in the polytene nuclei

**Figure 3.**
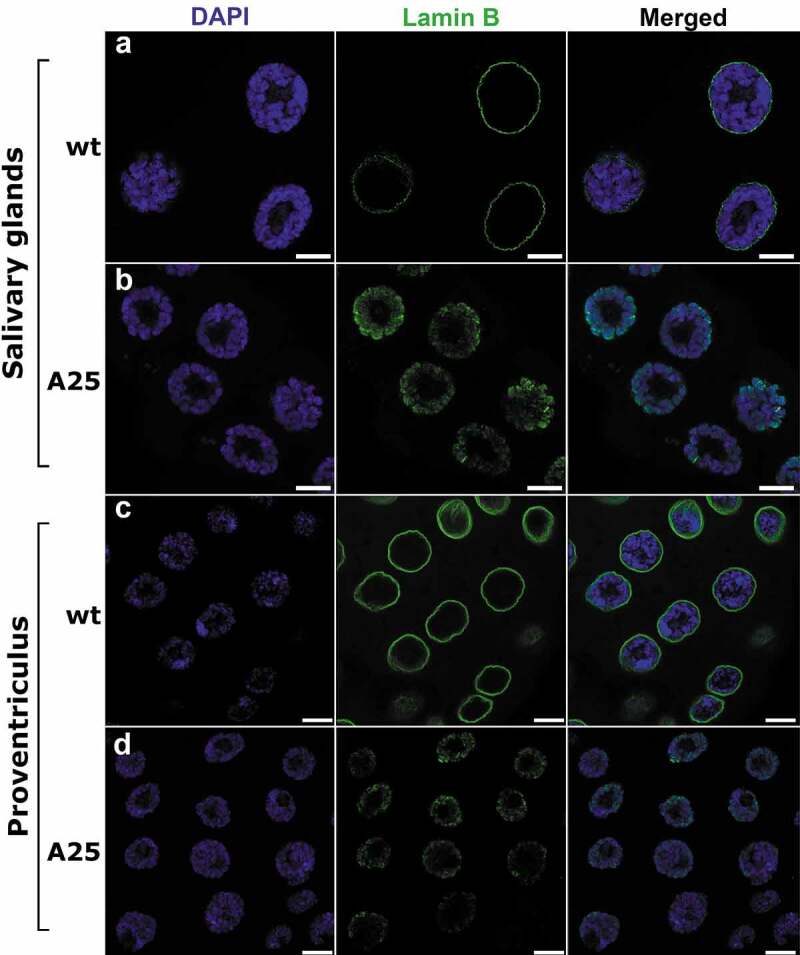
Whole-mount immunostaining of salivary gland (a, b) and proventriculus (c, d) nuclei of *wt* (a, c) and *Lam^A25^* (*A25*) mutant (b, d) *D. melanogaster* larvae. Chromatin (blue) is stained by DAPI. Lamin B (green) is stained by the specific antibody ADL67.10. Scale bar = 10 µm.

A previous study showed that lack of the CaaX box in *Lam^A25^* mutants causes the distribution of Lamin B throughout the nucleus in imaginal eye discs [12]. However, it was unclear whether mutant Lamin B still interacted with the nuclear envelope and whether it homogeneously occupied the entire nucleoplasm or preferentially associated with chromatin. The microscopy of small-sized nuclei from imaginal discs did not provide sufficient resolution to answer these questions [12]. We chose giant nuclei with polytene chromosomes of the salivary gland and the proventriculus to investigate the pattern of lamin distribution using high-resolution confocal microscopy. To visualize Lamin B, we immunostained the tissues of mutant and *wt* flies with two different antibodies. We found that the anti-Lamin B antibody ADL67.10 no longer formed a rim at the nuclear periphery in the *Lam^A25^* mutants as it does in *wt*. Instead, it occupied the internal nuclear space in the salivary gland (Figure 3(a,b)) and the proventriculus (Figure 3(c,d)). The distribution of Lamin B inside the nucleus was far from homogeneous as the ADL67.10 antibody was absent in the chromatin-free (DAPI-negative) nucleoplasm and colocalized with chromatin (**Figure S5**). Similarly, the majority of the ADL84.12 antibody abandoned the nuclear periphery and colocalized with the chromatin, although to a lesser extent than ADL67.10 especially in proventriculus nuclei (**Figure S6**).

### *Spatial repositioning of the chromatin in the* Lam^A25^ *mutant*

**Figure 4.**
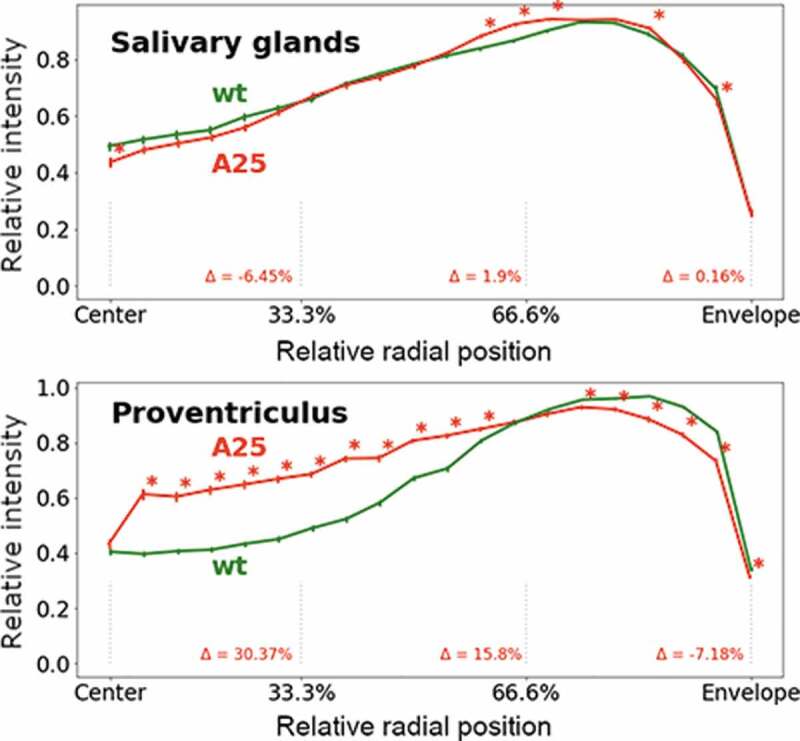
Radial fluorescence intensity of DAPI-stained chromatin in polytene nuclei of the salivary gland and the proventriculus in *D. melanogaster*. The X-axis is the relative position from the nuclear center to the periphery (0%-100%). The Y-axis is the intensity of DAPI fluorescence normalized by the maximum intensity in the nucleus. The green line represents *wt* data, the red line represents *Lam^A25^* mutant data. Error bars show standard deviation. Asterisks indicate 5% intervals with statistically significant *p-*values. Δ quantifies how the relative intensity in the *Lam^A25^* group changes in comparison with *wt* in each ⅓ interval of the X-axis.

To test whether the absence of Lamin B from the nuclear periphery causes spatial repositioning of chromatin, we measured the fluorescence intensity of DAPI along the nuclear radius divided by 5% intervals in the *wt* and *Lam^A25^* larvae. Quantification of the fluorescence intensity revealed that the effect of the mutation on the chromatin radial distribution differed between the salivary gland and the proventriculus nuclei (Figure 4). The majority of the 5% radial intervals (14 of 20) had no statistically significant differences in fluorescence intensity between the *wt* and *Lam^A25^* salivary gland nuclei. In contrast, a statistically significant difference in the fluorescence intensity between *wt* and *Lam^A25^* was observed in a majority (17 of 20) of the 5% radial intervals of the proventriculus nuclei. Importantly, the change in the DAPI fluorescence intensity was opposite for the central and peripheral chromatin of the proventriculus nuclei. We detected a statistically significant decrease in the fluorescence intensity in six (75%-100%) peripheral 5%-intervals in the proventriculus nuclei. A large and statistically significant increase in the fluorescence intensity is seen in 11 interior 5%-intervals (10%-60%) of the same tissue (Figure 4). Thus, the lack of Lamin B at the nuclear periphery is accompanied by a significant shift in the radial distribution of total chromatin from the nuclear periphery to the center in the proventriculus but not in the salivary gland cells.

**Figure 5.**
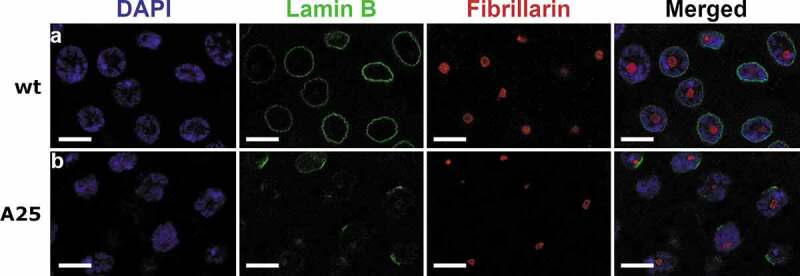
Immunostaining of proventriculus nuclei of *wt* and *Lam^A25^* (*A25*) mutant *D. melanogaster* larvae. Chromatin (blue) is stained by DAPI. Lamin B (green) is stained by the specific antibody ADL67.10. Nucleolus is stained by the fibrillarin antibody ab6785. Scale bar = 10 µm.

We also analyzed whether the measured increase of the DAPI fluorescence intensity in the nuclear center affects the chromatin position with respect to the nucleolus in the proventriculus nuclei of the *Lam^A25^* mutant. The nucleolus is a centrally located organelle surrounded by chromatin. When we performed immunostaining of the nucleolus with a fibrillarin antibody, we noticed an obvious chromatin-free space between the nucleolus and chromatin in *wt* nuclei (Figure 5). By comparison, the chromatin is almost immediately adjacent to the nucleolus in the *Lam^A25^* mutant, demonstrating that the chromatin position is shifted from the periphery toward the center in the proventriculus nuclei.

### *Localization of nuclear proteins and modified histones in the* Lam^A25^ *mutant*

**Figure 6.**
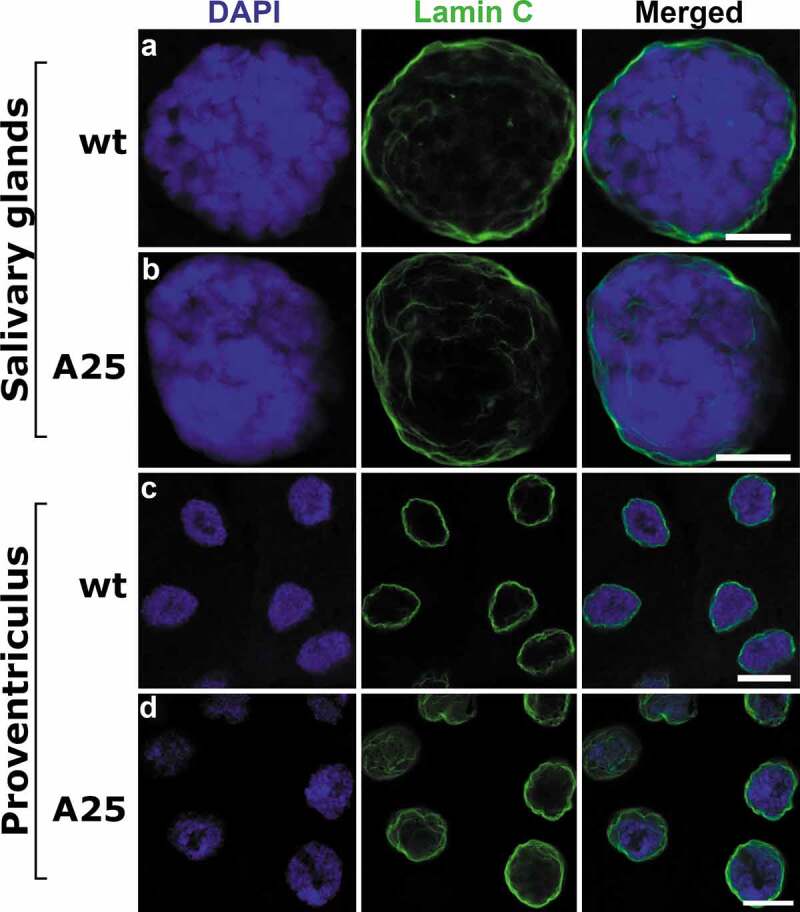
Localization of Lamin C in salivary gland and proventriculus nuclei from *wt* and *Lam^A25^ D. melanogaster* larvae. Chromatin (blue) is stained by DAPI. Lamin C (green) is stained by the specific antibody LC28.26. Scale bar = 10 µm.

Since the nuclear lamina of *D. melanogaster* consists of two lamins, Lamin B and Lamin C, we tested whether the nuclear localization of Lamin C is altered in the *Lam^A25^* mutant nuclei. Lamin C is normally located at the periphery of the polytenized nuclei in *D. melanogaster* [33]. Our immunostaining experiments with an anti-Lamin C antibody detected peripheral localization of this protein in the nuclei of both salivary glands and proventriculi in the homozygous *Lam^A25^* mutants as well as in *wt* (Figure 6). These results support the notion that Lamin C and Lamin B form independent meshworks of the *Drosophila* nuclear envelope [5,6]. Lamin C may ensure the mechanical properties of the nuclear envelope despite the dysfunctional Lamin B in the mutant nuclei.

Lamins are known to interact not only with the chromatin and nuclear membrane but also with integral proteins of the nuclear envelope 46. To investigate the effect of the lamin mutation on these proteins, we studied localization of the nuclear pore complex (NPC) proteins in *wt* and LamA25 mutant nuclei. We have not detected any obvious difference in NPC localization between the *wt* and mutant proventriculus nuclei (Figure S7). In the mutant salivary glands, the signal from anti-NPC antibody partially localizes both at the nuclear periphery and on polytene chromosomes repeating their banding pattern. This was not the case in *wt* salivary glands, where NPC localization strongly correlates with the nuclear envelope (Figure S7). We suggest that the mutant Lamin B, which is associated with polytene chromosomes, may attract a portion of NPCs toward the nuclear interior in salivary glands. Since the majority of the anti-NPC antibody labels the nuclear periphery in both proventriculus and salivary gland mutant nuclei, we conclude that the nuclear membrane is largely intact in the LamA25 larvae.

**Figure 7.**
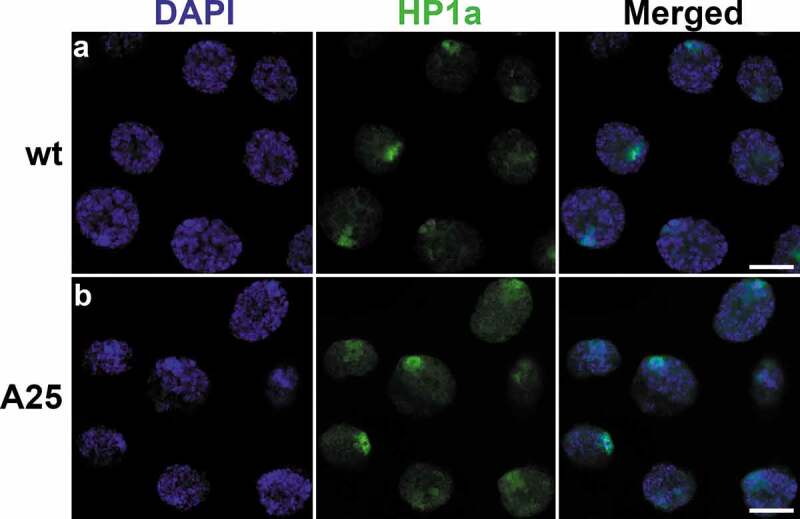
Localization of HP1a in proventriculus nuclei from *wt* (top) and *Lam^A25^* mutant (bottom) *D. melanogaster* larvae. Chromatin (blue) is stained by DAPI. HP1a (green) is stained by the specific antibody C1A9. Scale bar = 10 µm.

In *Drosophila*, there are several types of ‘silent’ chromatin recognized based on enrichment with specific nuclear proteins and modified histones [34,35]. Lamina-associated domains (LADs) are known as transcriptionally repressed regions largely corresponding to the ‘BLACK’ type of chromatin. *Drosophila* LADs are also slightly enriched in the H3K27me3 histone modifications, which typically mark the ‘BLUE’ chromatin associated with Polycomb group proteins [35,36]. The markers of the pericentromeric ‘GREEN’ chromatin, HP1a and H3K9me2, on the other hand, are not enriched in LADs in *D. melanogaster* Kc167 cells [35]. However, differentiated cell types in *Drosophila* (particularly neurons) have LAD-profiles that overlap HP1a/H3K9me2-profiles [36]. We wanted to test whether the structural organization or nuclear distribution of the ‘GREEN’ and ‘BLUE’ chromatin are affected by the *Lam^A25^* mutation. Since we observed a significant shift in the radial distribution of total chromatin from the nuclear periphery to the center in the *Lam^A25^* proventriculus, we performed immunostaining of this cell type with an HP1a antibody. Our study did not detect any obvious differences in the amount or localization of HP1a between the *wt* and *Lam^A25^* mutant flies (Figure 7). These data indicate that the *Lam^A25^* mutation can impact the nuclear distribution of euchromatin but does not affect the peripheral position or structure of the pericentromeric heterochromatin.

**Figure 8.**
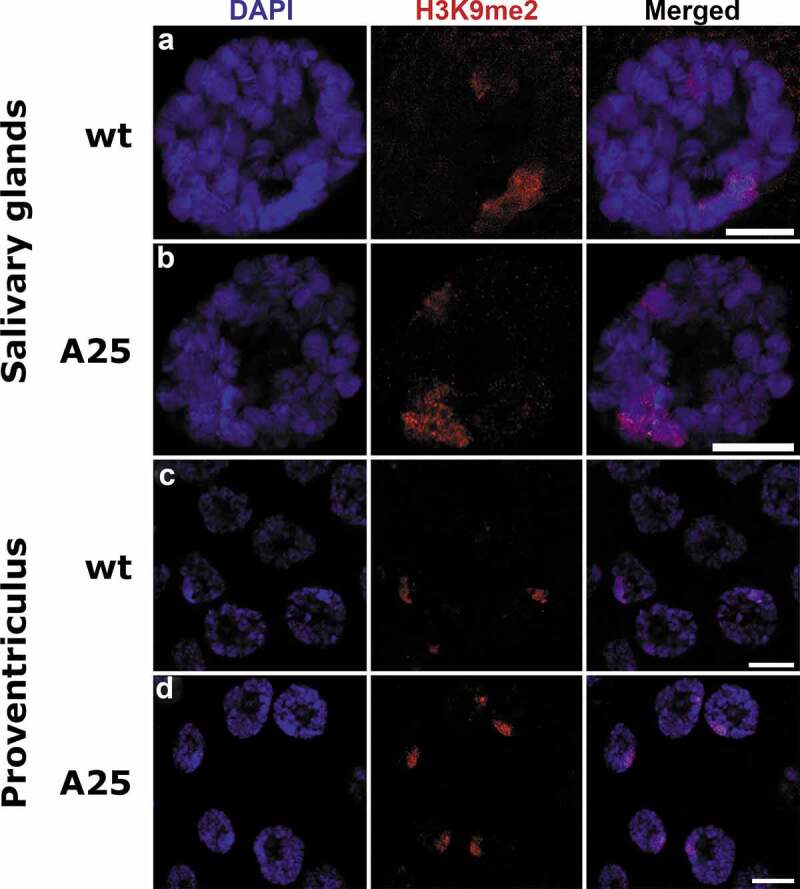
Localization of H3K9me2 in salivary gland and proventriculus nuclei from *wt* and *Lam^A25^ D. melanogaster* larvae. Chromatin (blue) is stained by DAPI. H3K9me2 (red) is stained by the specific antibody A-4035-025. Scale bar = 10 µm.

In addition to HP1a, we tested localization of another marker of the ‘GREEN’ chromatin, the H3K9me2 histone modification. We found similar peripheral nuclear positioning of H3K9me2 in salivary gland and proventriculus cells of both *wt* and mutant larvae (Figure 8). We conclude that the structural organization and localization of the chromocenter remain intact in the polytene nuclei of the *Lam^A25^* mutants.

Finally, since *Drosophila* LADs partially overlap with the ‘BLUE’ chromatin, we analyzed the staining pattern of the anti-H3K27me3 antibody in salivary gland and proventriculus cells of *wt* and *Lam^A25^* mutant larvae. We did not detect any obvious differences in the presence or amount of H3K27me3 between the *wt* and mutant nuclei indicating that the removal of the CaaX box does not affect the structural organization of ‘BLUE’ chromatin (**Figure S8**). We noticed that the H3K27me3 modification tends to associate with dense chromatin bands in both *wt* and mutant cells. Consistent with this observation, *Drosophila* LADs tend to colocalize with regions of densely packed chromatin [37,38].

## Discussion

In this work, we took advantage of the giant size of polytene chromosomes in salivary glands and proventriculus nuclei to conduct a high-resolution microscopic analysis of the nuclear architecture in *Lam^A25^ D. melanogaster* mutants. The linear organization of polytene and non-polytene chromosomes is identical as has been shown by the correlation between polytene chromosome bands and topologically associating domains (TADs) in diploid nuclei [39]. The larger size of polytene chromosome nuclei compared to regular diploid nuclei allowed us to increase the resolution of the microscopic analyses. Polytene chromosomes are best developed at the larval stage of fruit flies. However, the *Lam^A25^pr^1^/CyO;st^1^* line has markers for the identification of homozygous mutants at the adult stage. Therefore, we developed a crossing scheme for easier identification of homozygous *Lam^A25^* mutant larvae (Figure 1). We found that the banding pattern of polytene chromosomes from salivary glands of the homozygous *Lam^A25^* larvae has no detectable differences from that of *wt* larvae (**Figure S4**). We confirmed the identity of the *D. melanogaster* BSC line (#25092) by sequencing the entire mutant *Lam* gene (Figure 2). Our results agree with the previously reported location of this mutation and the predicted amino-acid sequence of the mutant protein [12].

A previous analysis of non-polytene nuclei in imaginal eye discs showed that Lamin B is no longer confined to the nuclear periphery in *Lam^A25^* mutants [12]. However, the precise pattern of intranuclear distribution of the mutant lamin and its effect on the chromatin architecture remained unclear. We showed that elimination of the CaaX box by the *Lam^A25^* frameshift mutation led to the abolishment of the peripheral location of Lamin B in the salivary gland and in the proventriculus nuclei (Figure 3**, S5, S6**). The effect of the removal of the CaaX box on lamin localization remained the same during the existence of the *Lam^A25^* mutant *Drosophila* line [12]. Lamin B was distributed in the internal space of the polytene mutant nuclei indicating that it cannot anchor to the nuclear envelope without the CaaX motif. This result suggests that the hydrophobic properties of the CaaX motif may be necessary for the attachment of Lamin B to the inner nuclear membrane [12,13]. In fact, when farnesylation of the CaaX motif was inhibited, the peripheral localization of lamin B2 decreased as its nucleoplasmic levels increased in human cells. However, other chemical properties of the CaaX box could also be responsible for the peripheral location of Lamin B. The strength of the CaaX box attachment to the lipid bilayer is rather weak and a second lipid-bilayer-binding moiety is required for stable association [13]. On the other hand, the CaaX box could be providing a nuclear localization signal used by inner membrane (LBR, LAP1/2, emerin, nurim, MAN1) or peripheral (otefin, YA, p-34, LBR-kinase) proteins [15] that may tether Lamin B to the nuclear membrane. In this case, other players could serve as physical intermediates between Lamin B and the inner nuclear membrane. Regardless of the mechanisms of interaction of Lamin B with the nuclear membrane, our data demonstrate that the CaaX box is necessary for the integrity of the lamina at the nuclear periphery.

Our analysis of high-resolution images of the salivary gland and proventriculus nuclei showed that mutant Lamin B is not homogeneously distributed in the nucleoplasm but is colocalized with polytene chromosomes. *In vitro* experiments established that the 425–473 and 572–622 amino acids of the *D. melanogaster* Lamin Dm0 sequence specifically bind chromosomes with an apparent dissociation constant of K_d_ ~ 1 µM [17]. The affected sequence of the *Lam^A25^* mutant protein starts after ^610^Gly and, thus, partially overlaps with the 572–622 sequence. This overlap may suggest a decreased strength of interaction between Lamin B and the chromatin. Apparently, the CaaX box is not required for this interaction, since the majority of Lamin B was associated with polytene chromosomes in the *Lam^A25^* salivary glands (**Figures S5, S6**). Other studies suggest that the ⍺-helical rod domain is necessary for the interaction of Lamin B with DNA or histones of the chromatin [3,16,40]. The ⍺-helical rod domain, which is intact in the *Lam^A25^* mutant, may be responsible for colocalization of Lamin B with polytene chromosomes. Future testing of multiple mutations that affect the ⍺-helical rod domain will map the region responsible for lamina–chromatin interaction in *D. melanogaster*. Immunostaining experiments will determine if other nuclear proteins can be mislocated due to mutations affecting the function of the ⍺-helical rod domain or the CaaX box of Lamin B.

Our work demonstrated that the removal of the Lamin B from the nuclear envelope by the *Lam^A25^* mutation can cause a significant shift in the radial distribution of chromatin from the periphery to the center of the proventriculus nucleus (Figure 4). This effect was confirmed by the visual observation of the diminished chromatin-free space between the nucleolus and chromatin in the mutant proventriculus (Figure 5). However, the chromatin radial distribution was similar between mutant and *wt* nuclei in salivary glands. We suggest that the observed difference between proventriculus and salivary gland nuclei is related to the different levels of polyteny between these two cell types. Salivary gland nuclei typically have high levels of polyteny reaching 1,024 C-2,048 C. Many other tissues in *D. melanogaster* larvae are also polytenized but generally not to the same high level as in salivary glands. For example, the prothoracic gland polyteny levels inferred from bandwidth measurements range between 256 C and 512 C [41]. The levels of polyteny positively correlate with the nuclear volumes, which are 18,850 µm^3^ and 5,783 µm^3^ for salivary glands and prothoracic glands, respectively [41]. In our study, the average nuclear diameters were 26.8 µm and 25.7 µm in the *wt* and mutant salivary gland cells but were only 11.1 µm and 11.7 µm in the *wt* and mutant proventriculus cells (**Table S2**). Accordingly, the average nuclear volumes in our study for *wt* salivary glands and proventriculi were 10,079 µm^3^ and 716 µm^3^, respectively. Therefore, we conclude that the levels of chromosome polytenization in our preparations were no greater than 1,024 C for salivary glands and substantially less than 256 C for proventriculi.

The difference in the levels of polyteny can explain the cell type-specific effect of the *Lam^A25^* mutation of chromatin architecture in several ways. First, highly polytenized chromosomes in salivary glands have bands of intercalary heterochromatin that fail to complete replication in the S phase of the endocycle and stay underreplicated, leading to special morphological characteristics of these regions such as breaks, constrictions, and ectopic contacts [42]. These properties allow chromosomes to more easily bend in these regions and form associations with each other and with other structures. It has been shown that ectopic chromatin fibers resulting from undereplication help intercalary heterochromatin to physically attach to the nuclear surface [41,43]. Of 48 chromosome-nuclear envelope attachment regions identified in salivary gland nuclei, 34 belong to intercalary heterochromatin, 11 display some properties of intercalary heterochromatin by being late replicating regions, and only 3 belong to euchromatin [44]. In nonpolytene diploid nuclei, the chromosome-nuclear envelope attachments occur *via* specific interactions between 412 LADs and Lamin B [45]. Comparison of these LADs with regions of intercalary heterochromatin in salivary glands identified substantial overlap between them. For example, as many as 64% of LADs are located within heterochromatin bands and up to 70% of bands replicating at late stage 3 overlap with LADs [38]. It is possible that in highly polytenized nuclei, the properties conferred by undereplication of the intercalary heterochromatin (such as ‘stickiness’ of ectopic fibers and bending at the weak points) play a more important role in tethering chromosomes to the nuclear periphery than the affinity between LADs and lamina. In this case, the chromosome-nuclear envelope attachments may no longer rely on Lamin B but may occur *via* other peripheral proteins such as Lamin C. As a result, polytene chromosomes may still be attached to the nuclear envelope in salivary glands of the *Lam^A25^* mutants. In contrast, the regions of intercalary heterochromatin may not play such an important role in tethering chromosomes to the periphery in proventriculus nuclei where the level of polyteny is very low. Interestingly, the level of heterochromatin underreplication is lower in ovarian pseudonurse cells than in salivary gland cells of *D. melanogaster* [46]. In agreement with this observation, the percentage of chromosome regions located at the nuclear periphery was typically higher in salivary gland cells than in ovarian nurse cells of mosquitoes [47]. Second, it has been shown that chromosomes occupy a significantly greater fraction of the nuclear volume in salivary glands (34%) than in other tissues with lower levels of polyteny [41]. Even within prothoracic glands, the volume fraction occupied by chromosomes in the more highly polytenized nuclei was significantly higher than in the smaller ones, 20% for 512 C and 13% for 256 C. Therefore, highly polytenized chromosomes may have less free space for the movement that may be necessary for a change in chromatin radial distribution. Finally, the giant salivary gland chromosomes may have significantly reduced mechanical flexibility making them less responsive to factors influencing the nuclear architecture. For example, Hi-C experiments found no reproducible, long-range interactions in polytene chromosomes of salivary glands but detected specific distant chromosomal contacts in *Drosophila* embryos [39]. Also, the number of LADs in diploid nonpolytene nuclei is 10 fold higher than the number of polytene chromosome regions attached to the nuclear envelope [44,45]. It is possible that all these special properties of the highly polytenized chromosomes contribute to preserving the *wt* chromatin architecture in the salivary gland nuclei of the *Lam^A25^* mutants. Future studies of other cell types (including nonpolytene diploid) will clarify the role of Lamin B in spatial genome organization.

It has been shown that B-type and A-type lamins have different structural roles in nuclear periphery: the B-type lamin meshwork is responsible for the organization of chromatin whereas the A-type meshwork governs the stiffness of the nucleus and maintains the nuclear shape [48]. Our immunostaining experiments showed that *Lam^A25^* mutant nuclei have intact Lamin C at their periphery (Figure 6). Additionally, we did not detect any misshapen nuclei in *Lam^A25^* mutants. Our data suggest that Lamin B is dispensable for the preservation of normal nuclear geometry in the presence of Lamin C.

A study of adult brains in *Lam^A25^ Drosophila* mutants detected significantly reduced levels of the heterochromatin marks H3K9me2 and HP1a (by western analysis) as well as a substantial loss of chromocenter staining in neurons (by immunostaining), suggesting that dysfunctional Lamin B disrupts chromatin architecture [22]. This observation is consistent with the fact that LAD-profiles in both the chromosome arms and pericentromeric regions in *Drosophila* neurons display striking concurrence with HP1a and H3K9me2 [36]. This overlap is cell type-specific, as it is present to a lesser extent in glia and the fat body [36] and absent in Kc167 cells [35]. Unlike neurons [22], we did not observe any reduction in the intensity of immunostaining with H3K9me2 or HP1a antibodies in mutant polytene nuclei. Also, both H3K9me2 and HP1a preserve a peripheral nuclear location in salivary glands and proventriculi of the *Lam^A25^* flies (Figure 7, 8). These data suggest that the interaction of the pericentromeric ‘GREEN’ chromatin with the envelope in polytene nuclei is not dependent on Lamin B. A *Drosophila* pericentromeric sequence, which contains matrix/scaffold attachment regions (M/SARs), binds specifically (in comparison to the *Escherichia coli* DNA) to authentic as well as bacterially expressed polymers of Lamin Dm0 *in vitro* [49]. However, M/SAR sequences can physically interact not only with B-type lamins but also with A-type lamins and the structurally related proteins desmin and NuMA of rat liver nuclear envelopes [50]. It is possible that *Drosophila* pericentromeric repeats and/or associated proteins can bind to various components of the nuclear envelope preserving the peripheral position of heterochromatin in the *Lam^A25^* mutants. Underreplication of the pericentromeric heterochromatin in polytene cells may provide additional properties that help anchor the chromocenters to the nuclear envelope. Also, the staining pattern with the anti-H3K27me3 antibody was similar in the *wt* and *Lam^A25^* mutant salivary glands and proventriculi **(Figure S8**) indicating that the relocation of Lamin B from the periphery to the nuclear interior does not affect the structural organization of the ‘BLUE’ type of chromatin in these cell types.

**Figure 9.**
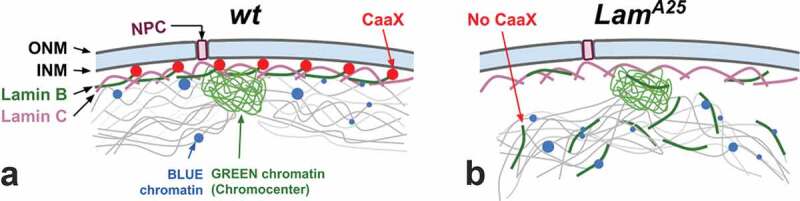
Scheme demonstrating the role of the CaaX box of Lamin B in nuclear organization. (a) Organization of the nuclear periphery in the *wt* nucleus. (b) The effect of CaaX box removal on nuclear architecture in the *Lam^A25^* mutant. Chromatin fibers (thin gray lines) are shown interacting with Lamin B (thick green lines). CaaX boxes (red circles) are shown interacting with the inner nuclear membrane (INM) in *wt* flies. ONM stands for the outer nuclear membrane. The scheme reflects data obtained from *Drosophila* nuclei with low levels of polyteny found in proventriculus.

Taken together, our results demonstrate that removal of the CaaX box by the *Lam^A25^* mutation disrupts the interaction of Lamin B with the nuclear membrane but not with the chromatin (Figure 9). In agreement with this finding, a recent study reported that CaaX-motif-deleted modified Lamin B is stably associated with chromatin *via* the Ig-fold domain inside of the *Drosophila* polytene nuclei [51]. However, it is still unclear whether Lamin B binds to the same LADs in the mutant flies as it does in the *wt* flies, or if the mutant protein occupies new chromatin sites. A DamID experiment on *Lam^A25^* mutant cells could answer this question. Our data also indicate that tethering of the chromatin to the nuclear periphery is impaired in nuclei with the mutant Lamin B as is clearly seen in the proventriculus cells. These results are in agreement with recent knockdown experiments that demonstrated the role of Lamin B in holding peripheral chromatin in close proximity to the nuclear membrane in a *Drosophila* cell line [18]. Interestingly, Lamin B may play a lesser role in chromatin organization in mammalian cells. Recent studies demonstrated that depletion of Lamin B (as well as LAP2a, Emerin, CTCF, and Cohesin) had little effect on chromatin dynamics in mouse cells, while depletion of Lamin A or BAF strikingly increased interphase chromatin diffusion in mouse embryonic fibroblasts [52,53].

The *Lam^A25^* adult flies are viable, although sterile (our data), with reduced survival [22], meaning that the mutant protein still performs some of its essential functions. Several genes (*uif, nvd, CG15115*), which can be affected by heterochromatin relaxation [54], expressed at higher levels in *Lam^A25^* mutant fly heads, suggesting a role of Lamin B in epigenetic regulation of gene expression [22]. In addition, *Lam^A25^* homozygotes had abnormal accumulation of polyadenylated RNA in brains, suggesting that Lamin B dysfunction causes aberrant RNA trafficking and localization [23]. Detailed studies of interactions between Lamin B domains and nuclear compartments (chromatin, nuclear membrane, nucleolus) will increase our understanding of the interplay between the spatial genome organization and the orchestration of gene activity in different cell types.

## Supplementary Material

Supplemental Material

## Data Availability

The data that support the findings of this study are openly available in figshare.com at https://figshare.com/articles/Salivary_gland_and_proventriculus_radial_intensity/11299010, reference number DOI:10.6084/m9.figshare.11299010.
