# Supplementary material for: Reorganization of the nuclear architecture in the Drosophila melanogaster Lamin B mutant lacking the CaaX box
Source: Nucleus. 2020 Sep 22;11(1):283–98. doi: 10.1080/19491034.2020.1819704 (PMC7529411; doi:10.1080/19491034.2020.1819704)
Supplement: Supplemental Material [file KNCL_A_1819704_SM3994.docx]

# Supplementary material

This file contains

- Supplementary Tables S1-S2

- Supplementary Figures S1-S8

**Table S1.** Primer used for PCR and Sanger sequencing of the *Lamin B* gene.

| **Name** | **Forward primer** | **Reverse primer** | **Lam_start** | **Lam_stop** | **Tm (**ºC**)** |
| --- | --- | --- | --- | --- | --- |
| **Lam1** | TCTTTTATCTAGCGCGGCGT | CATCGCTTTCCACTCAAGCG | 10 | 530 | 59.9/59.9 |
| **Lam2** | ATTTGCCTCCACGCCCATAA | GTTCTGCAGCTCCACCTTCT | 442 | 1032 | 60.03/59.96 |
| **Lam3** | ATGTGCGTGTGTGTGAGTGA | CCCTAGCTGTGTCATCGAGC | 528 | 1213 | 60.18/60.25 |
| **Lam4** | TGGAGCTGCAGAACCTGAAC | CAGCTCCTTTAGCGCCTCAT | 1019 | 1671 | 60.25/60.18 |
| **Lam4-5** | CTCTGGGAGGAGAACGAGGA | CAGCGACTGCTTCAACTTGG | 1246 | 1899 | 60.03/59.76 |
| **Lam5** | GCAATGTCCGCATGTACGAG | GCATTGGCTTGCTCCAGTTC | 1559 | 2105 | 59.70/60.11 |
| **Lam6** | TCGCGATGAAATCCAGTCCC | GCACTGGCGGACACATAGTA | 1941 | 2566 | 60.18/59.83 |
| **Lam7** | CTGGATTTGGAAATCGCCGC | CGCACTTCTCGTTTGACTGC | 2334 | 3018 | 60.25/60.11 |
| **Lam7-8** | AGGGCAACGTGGAGATCAAG | CTGTACCTCGGTGTGTGTGT | 2569 | 3140 | 60.04/59.61 |
| **Lam8** | CCCAACACACATCCTCCTCC | TTTCAAAGTTCTTGCCGCCG | 2905 | 3431 | 60.04/59.97 |
| **Lam9** | ACACACACACCGAGGTACAG | TGGTTGTCACTTCCGTAGGC | 3121 | 3745 | 59.61/59.97 |
| **Lam10** | AACCAGTCGACGTCAGCAAA | TGTGGGCGTTGCATTTGTTT | 3517 | 4045 | 60.18/59.82 |

**Table S2.** Nuclear diameters in the wild-type and mutant salivary gland and proventriculus cells.

| **Cell type** | **Diameter of salivary gland nuclei (µm)** | | **Diameter of proventriculus nuclei (µm)** | |
| --- | --- | --- | --- | --- |
| **Phenotype** | *Lam[A25]* | *Wild-type* | *Lam[A25]* | *Wild-type* |
|  | 22.86 | 22.95 | 11.93 | 12.91 |
|  | 19.67 | 27.68 | 10.84 | 12.70 |
|  | 21.46 | 29.78 | 11.53 | 13.58 |
|  | 18.17 | 26.17 | 10.57 | 12.36 |
|  | 23.47 | 24.15 | 10.94 | 11.00 |
|  | 19.99 | 24.92 | 9.84 | 10.03 |
|  | 31.03 | 24.53 | 11.81 | 12.25 |
|  | 21.28 | 28.15 | 10.24 | 12.49 |
|  | 22.85 | 29.79 | 10.05 | 10.13 |
|  | 27.70 | 29.46 | 10.64 | 9.37 |
|  | 25.39 | 25.28 | 11.25 | 10.07 |
|  | 29.63 | 26.23 | 11.64 | 10.31 |
|  | 25.51 | 28.86 | 10.42 | 9.43 |
|  | 24.69 | 27.64 | 9.63 | 12.73 |
|  | 25.87 | 28.44 | 9.22 | 13.00 |
|  | 23.77 | 23.50 | 9.88 | 13.28 |
|  | 27.87 | 30.77 | 11.97 | 10.64 |
|  | 29.47 | 22.70 | 9.67 | 9.75 |
|  | 33.80 | 21.96 | 10.05 | 12.12 |
|  | 30.40 | 23.36 | 9.10 | 9.20 |
|  | 26.83 | 22.35 | 9.10 | 9.61 |
|  | 27.10 | 29.54 | 16.16 | 11.09 |
|  | 24.42 | 29.31 | 12.56 | 10.79 |
|  | 20.60 | 30.61 | 11.07 | 8.67 |
|  | 23.53 | 28.55 | 10.53 | 8.27 |
|  | 22.59 | 28.16 | 11.60 | 10.10 |
|  | 32.08 | 27.53 | 12.83 | 10.59 |
|  | 26.47 | 29.09 | 13.29 | 8.99 |
|  | 31.00 | 30.21 | 11.90 | 9.60 |
|  | 28.07 | 19.79 | 16.76 | 10.49 |
|  | 27.87 | 34.99 | 13.05 | 8.24 |
|  | 25.19 | 33.82 | 9.69 | 11.03 |
|  | 24.78 | 32.72 | 11.13 | 8.43 |
|  | 24.67 | 31.03 | 10.80 | 7.72 |
|  | 26.80 | 26.79 | 14.31 | 8.25 |
|  | 22.18 | 26.47 | 17.79 | 16.03 |
|  | 26.30 | 25.66 | 18.78 | 12.56 |
|  | 27.63 | 25.64 | 14.69 | 15.80 |
|  | 24.16 | 26.20 | 16.49 | 14.40 |
|  | 30.80 | 26.79 | 13.43 | 12.17 |
|  | 29.78 | 23.19 | 12.10 | 10.04 |
|  | 26.44 | 22.95 | 14.08 | 11.16 |
|  | 28.07 | 27.68 | 11.31 | 5.65 |
|  | 22.86 | 29.78 | 11.28 | 9.17 |
|  | 19.67 | 26.17 | 16.18 | 14.32 |
|  | 21.46 | 24.15 | 16.13 | 8.06 |
|  | 18.17 | 24.92 | 13.66 | 11.88 |
|  | 23.47 | 24.53 | 14.21 | 13.39 |
|  | 19.99 | 28.15 | 13.13 | 13.38 |
|  | 31.03 | 29.79 | 12.69 | 12.73 |
|  | 21.28 | 29.46 | 10.06 | 13.20 |
|  | 22.85 | 25.28 | 11.35 | 13.67 |
|  | 27.70 | 26.23 | 10.53 | 12.58 |
|  | 25.39 | 28.86 | 10.11 | 11.11 |
|  | 29.63 | 27.64 | 10.33 | 11.84 |
|  | 25.51 | 28.44 | 9.00 | 12.90 |
|  | 24.69 | 23.50 | 9.29 | 14.67 |
|  | 25.87 | 30.77 | 9.15 | 14.11 |
|  | 23.77 | 22.70 | 11.04 | 11.05 |
|  | 27.87 | 21.96 | 11.09 | 10.88 |
|  | 29.47 | 23.36 | 9.39 | 8.55 |
|  | 33.80 | 22.35 | 8.11 | 9.92 |
|  | 30.40 |  | 10.14 | 8.41 |
|  | 26.83 |  | 7.72 | 8.23 |
| **Average** | **25.69** | **26.83** | **11.71** | **11.11** |
| **Standard deviation** | **3.75** | **3.19** | **2.39** | **2.16** |


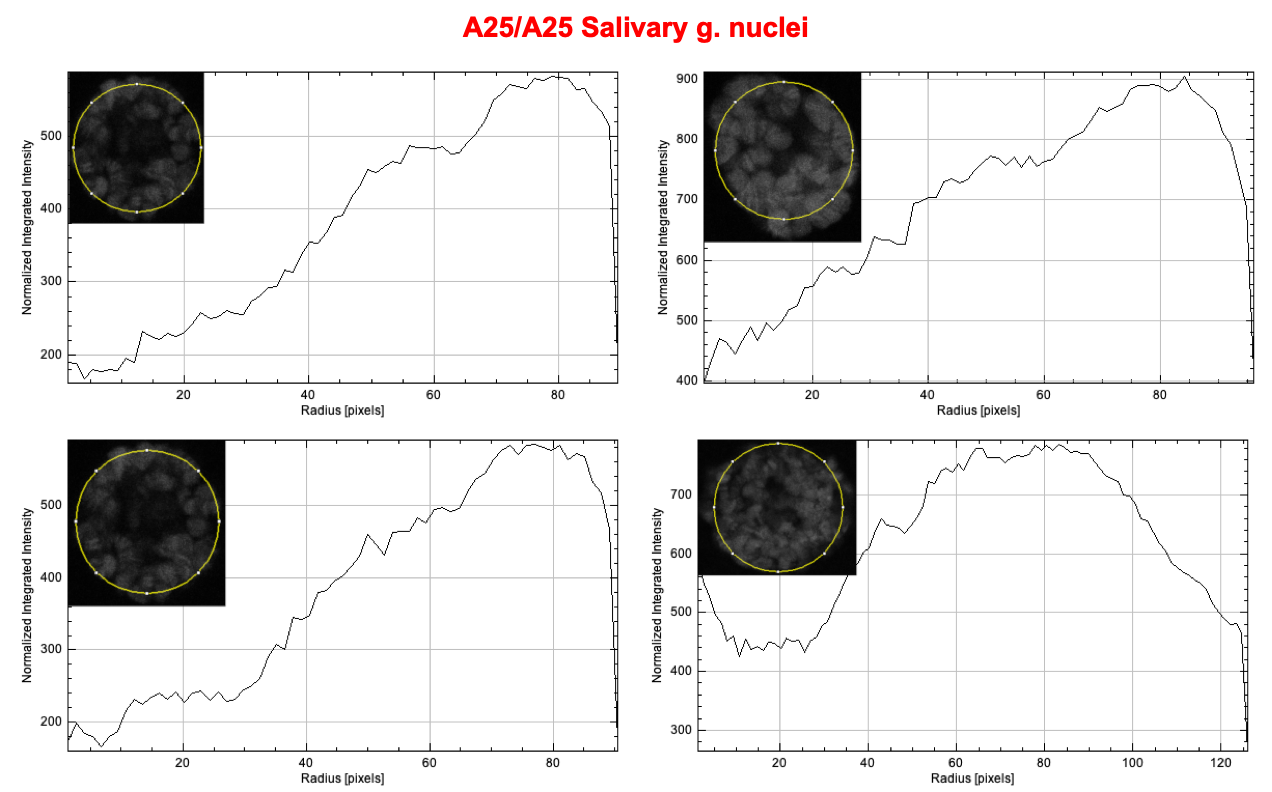


**Figure S1.** The radial fluorescence intensity of the chromatin in four individual polytene nuclei of salivary gland of the *D. melanogaster Lam^A25^* homozygous line. The X axis is the relative position from the nuclear center to the periphery (0%-100%). The Y axis is the intensity of chromatin fluorescence normalized by the maximum intensity in the nucleus.


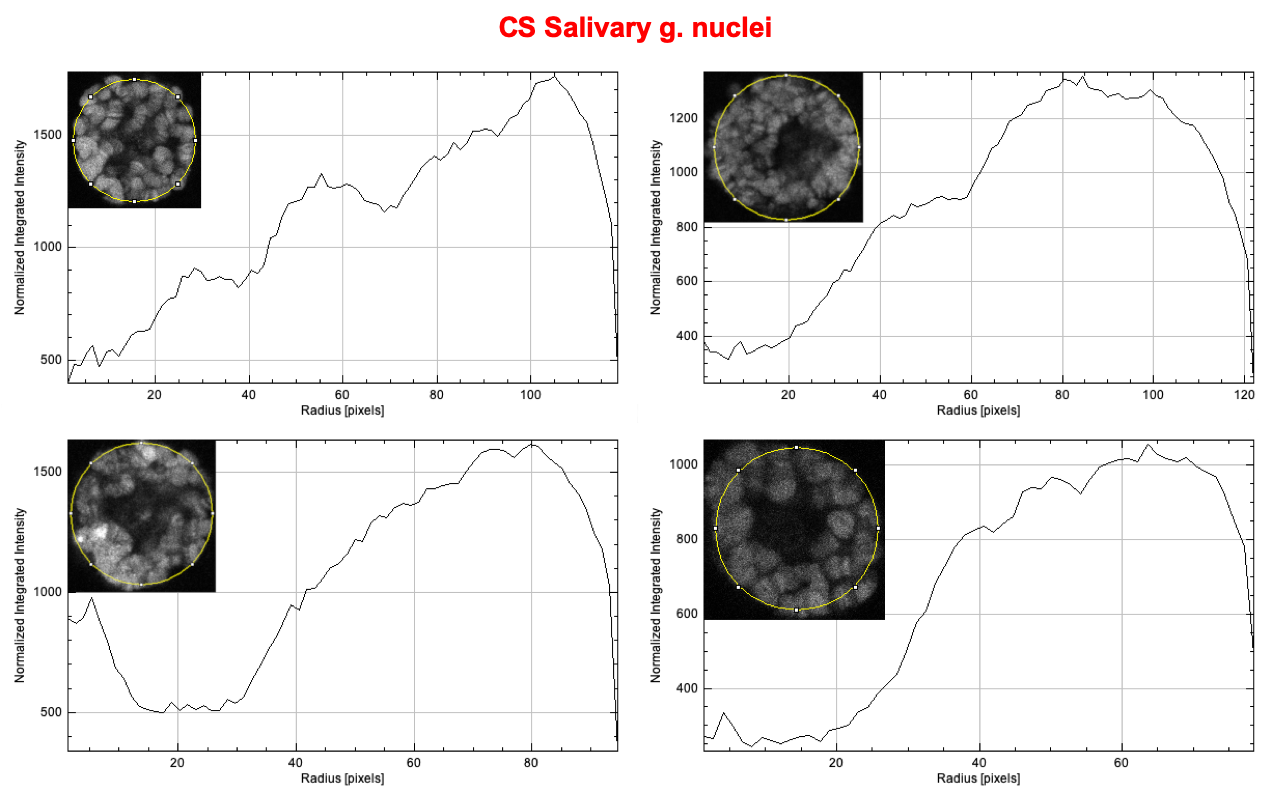


**Figure S2.** The radial fluorescence intensity of the chromatin in four individual polytene nuclei of the salivary gland of the *D. melanogaster* Canton-S line. The X axis is the relative position from the nuclear center to the periphery (0%-100%). The Y axis is the intensity of chromatin fluorescence normalized by the maximum intensity in the nucleus.


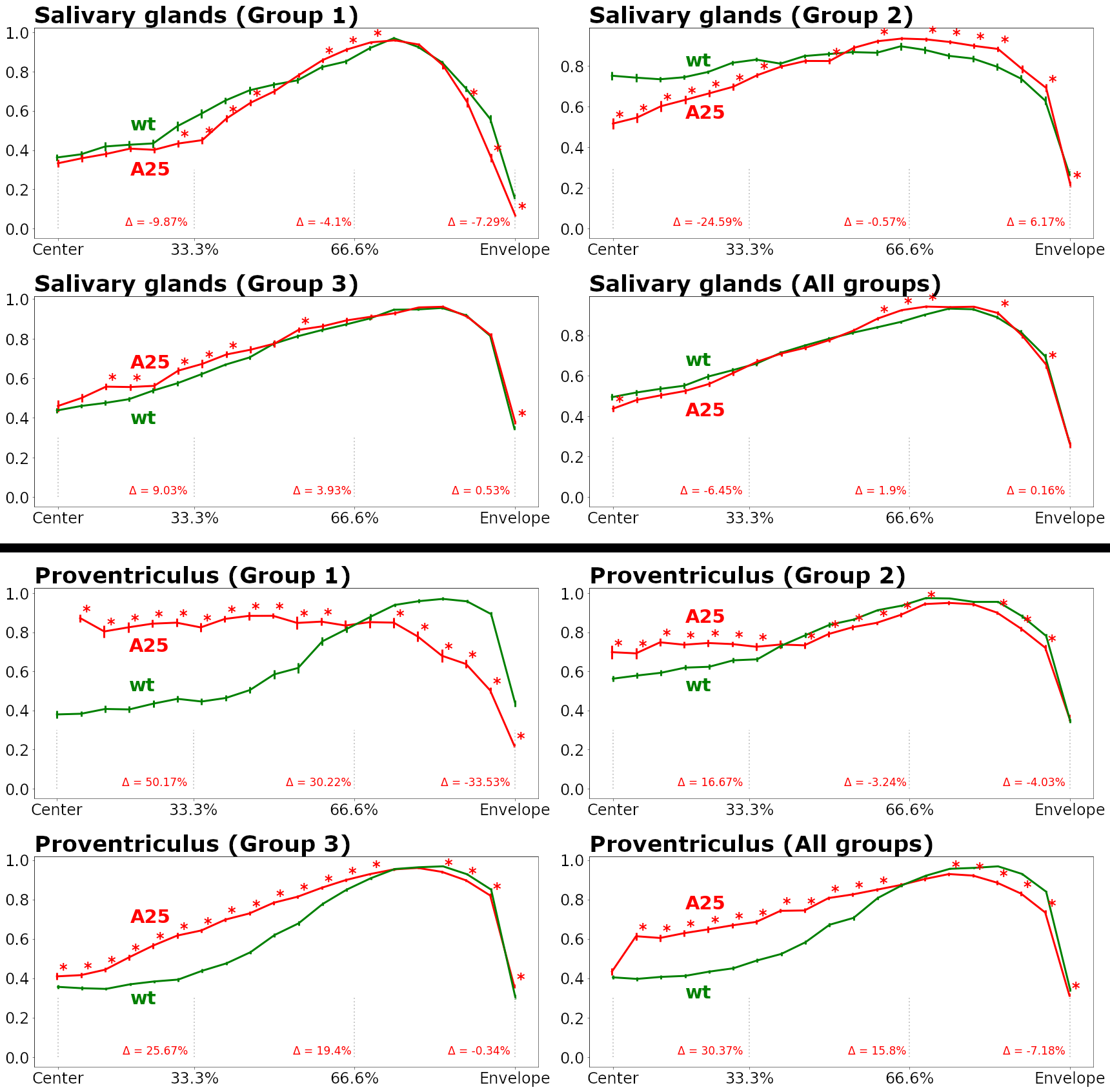


**Figure S3.** The radial fluorescence intensity of the chromatin in polytene nuclei of salivary gland (top panel) and proventriculus (bottom panel) in three experimental groups of *D. melanogaster*. The X axis is the relative position from the nuclear center to the periphery (0%-100%). The Y axis is the intensity of chromatin fluorescence normalized by the maximum intensity in the nucleus. The green line represents wild-type data, the red line represents *Lam^A25^* data. Error bars show standard deviation. Asterisks indicate 5% intervals with statistically significant *p-*values.


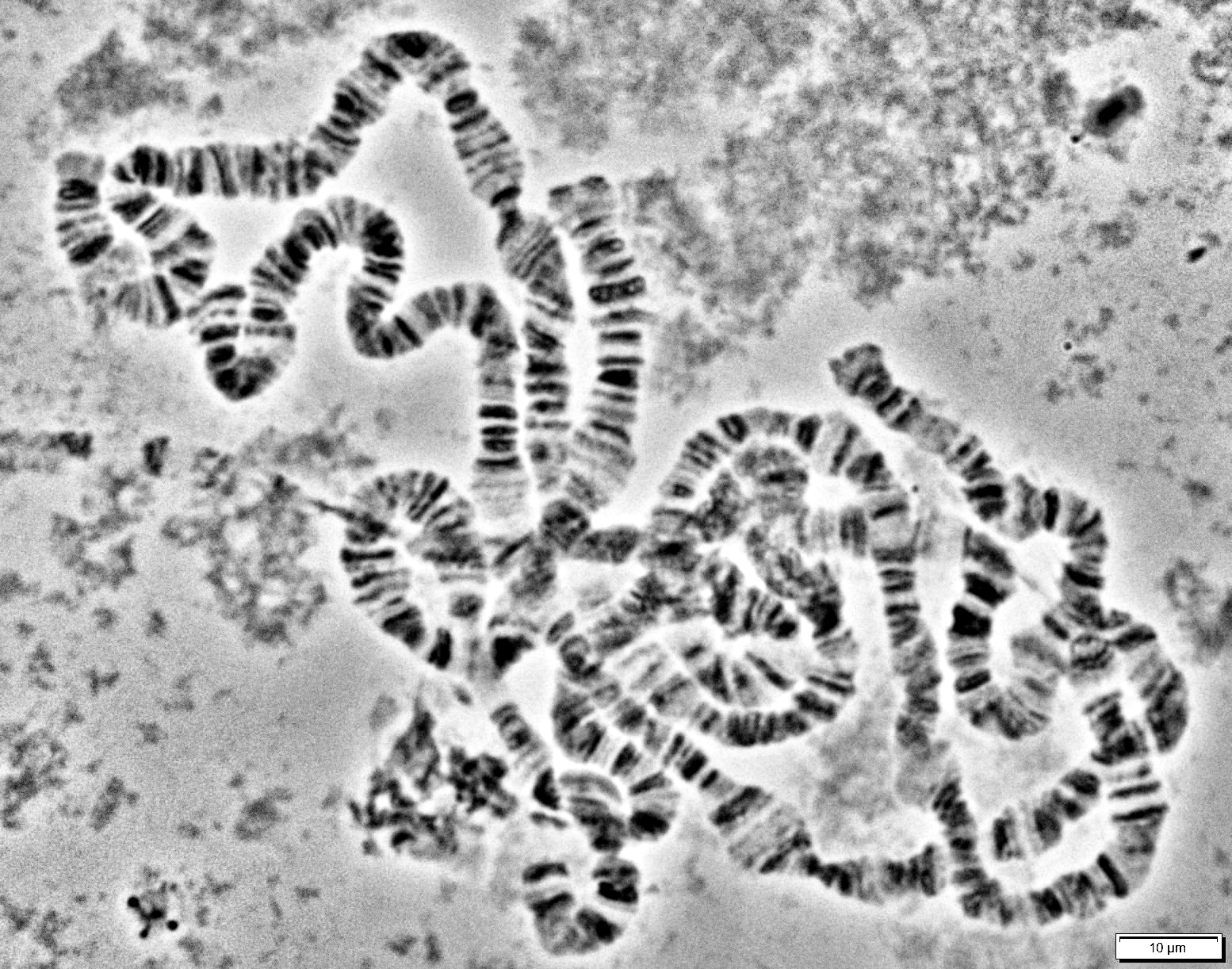


**Figure S4.** Salivary gland polytene chromosomes from homozygous *Lam^A25^* mutant *D. melanogaster* larvae. Scale bar = 10 µm.

##
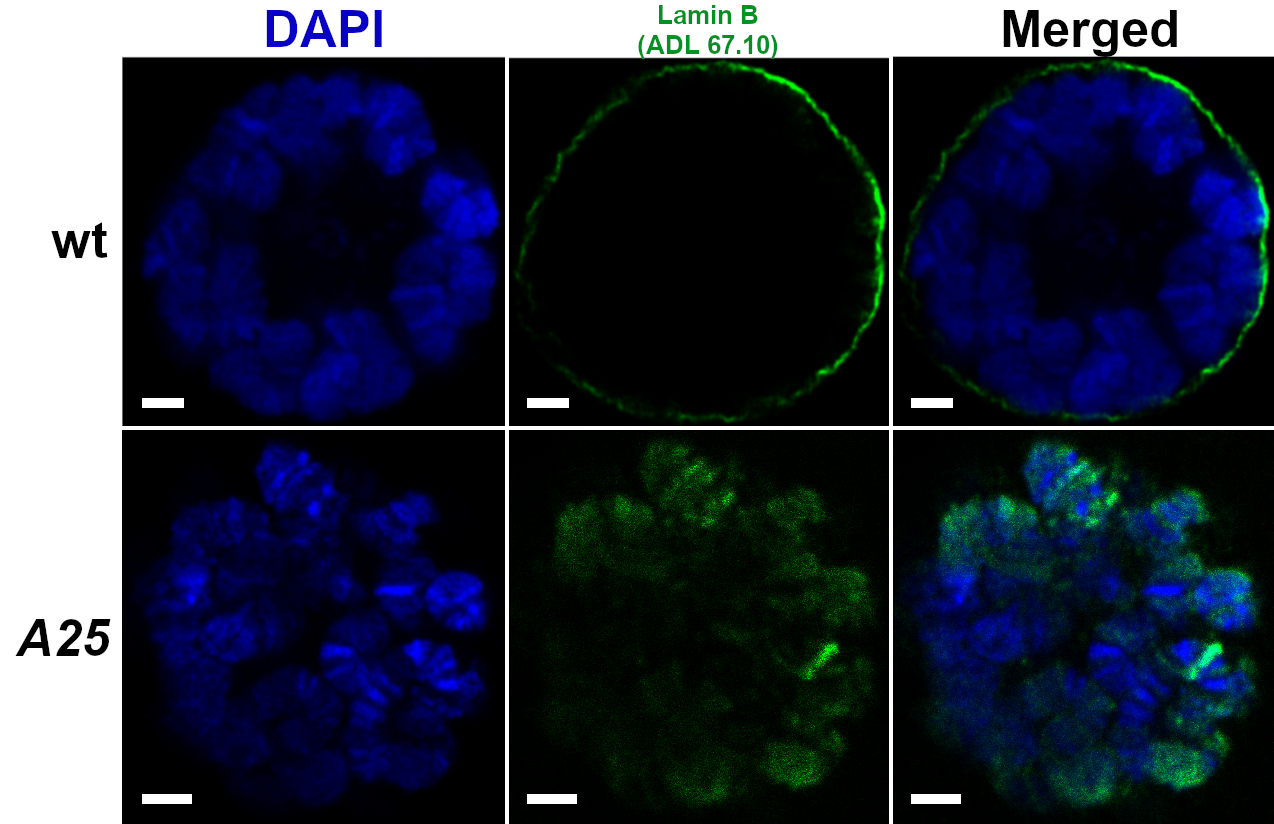


**Figure S5.** High-magnification images of salivary gland nuclei in a wild-type (*wt*) and *Lam^A25^* (*A25*) *D. melanogaster* mutant. Chromatin (blue) is stained by DAPI. Lamin B (green) is stained by the specific antibody ADL67.10. Scale bar = 2 µm.


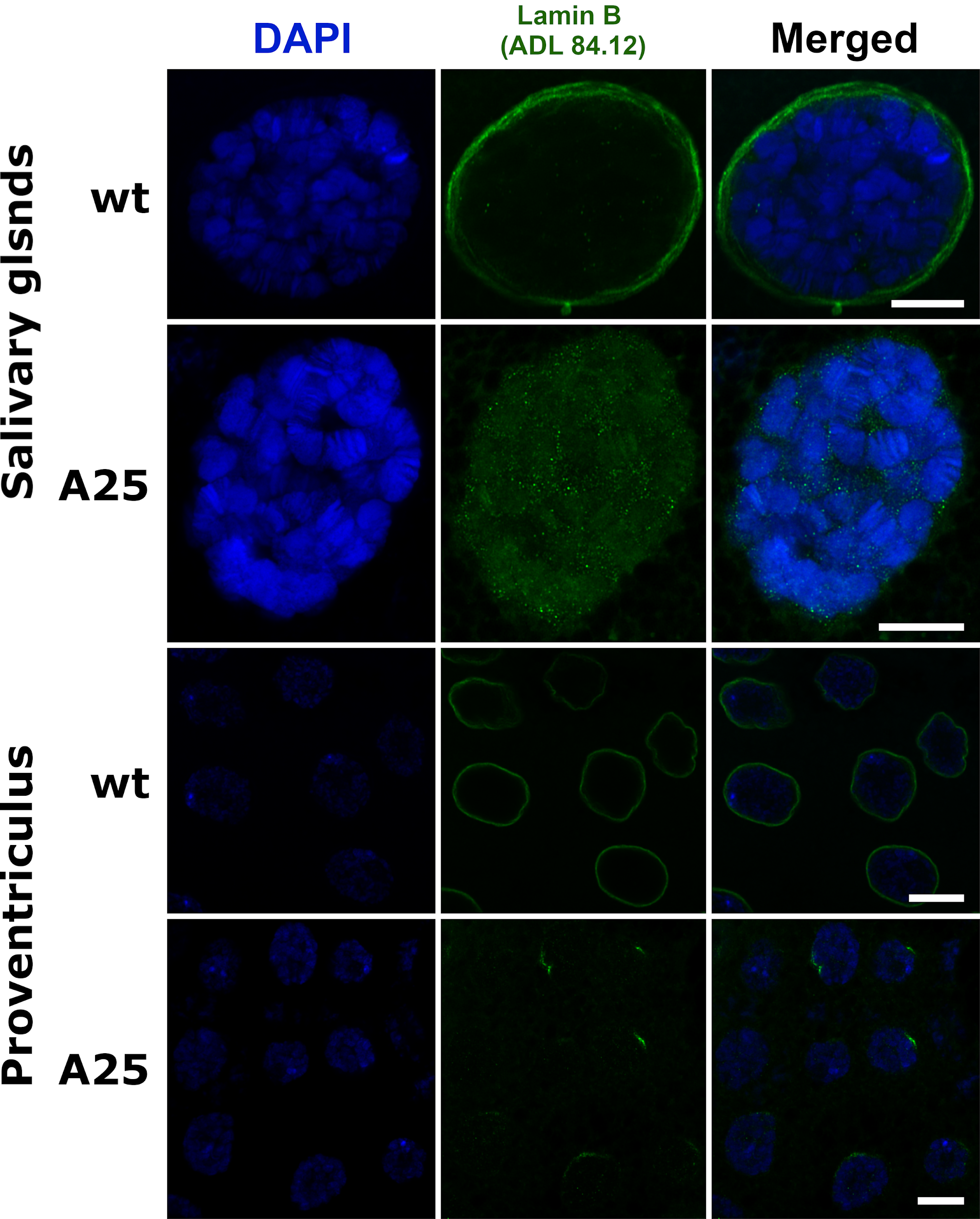


**Figure S6**. Localization of ADL84.12 antibody in salivary gland and proventriculus nuclei from wild-type (top) and *Lam^A25^* mutant.


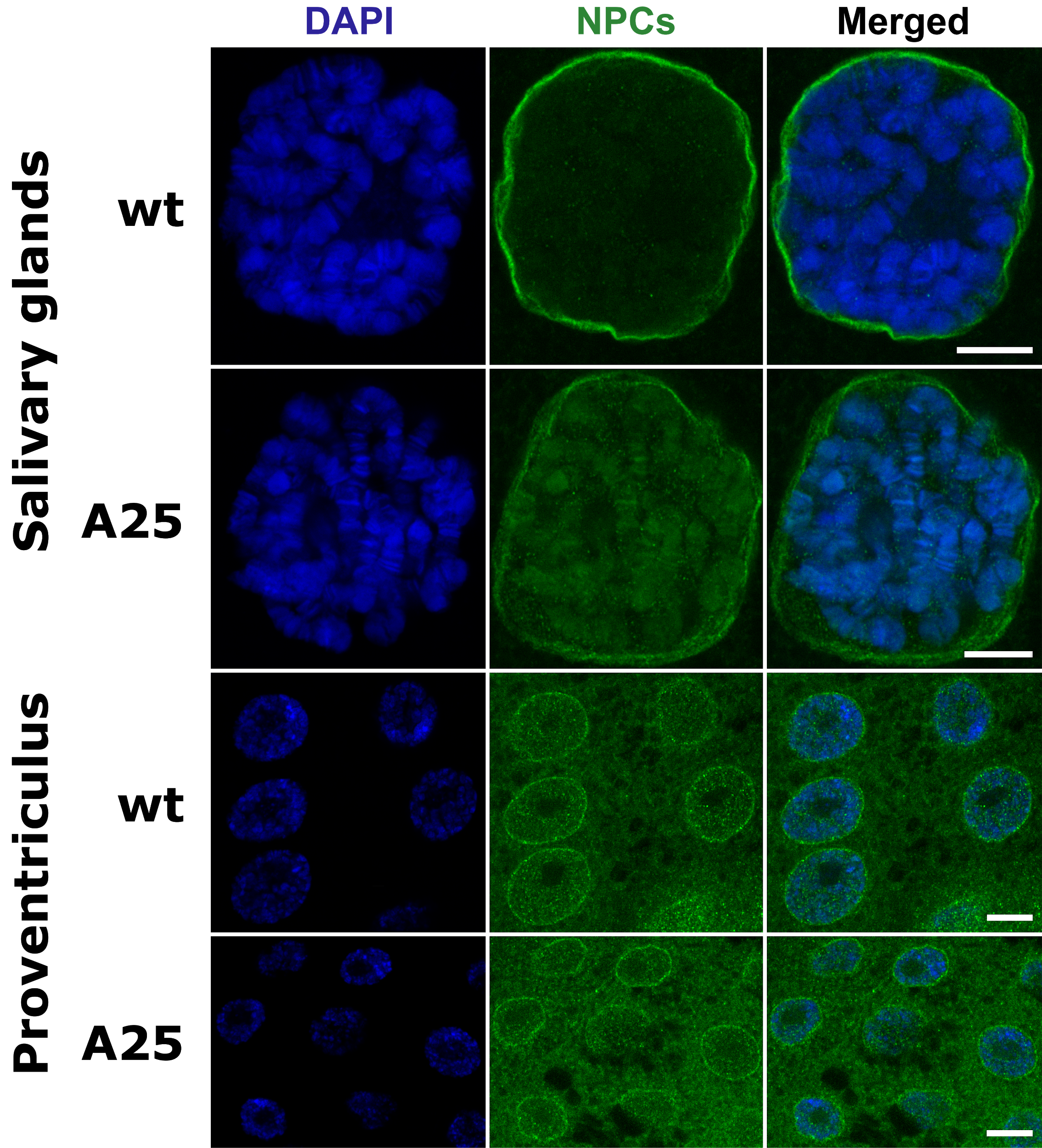


**Figure S7**. Localization of NPCs in salivary gland and proventriculus nuclei from *wt* and *Lam^A25^* *D. melanogaster* larvae. Chromatin (blue) is stained by DAPI. NPCs (green) are stained by the specific antibody 1515-NPC. Scale bar = 10 µm.


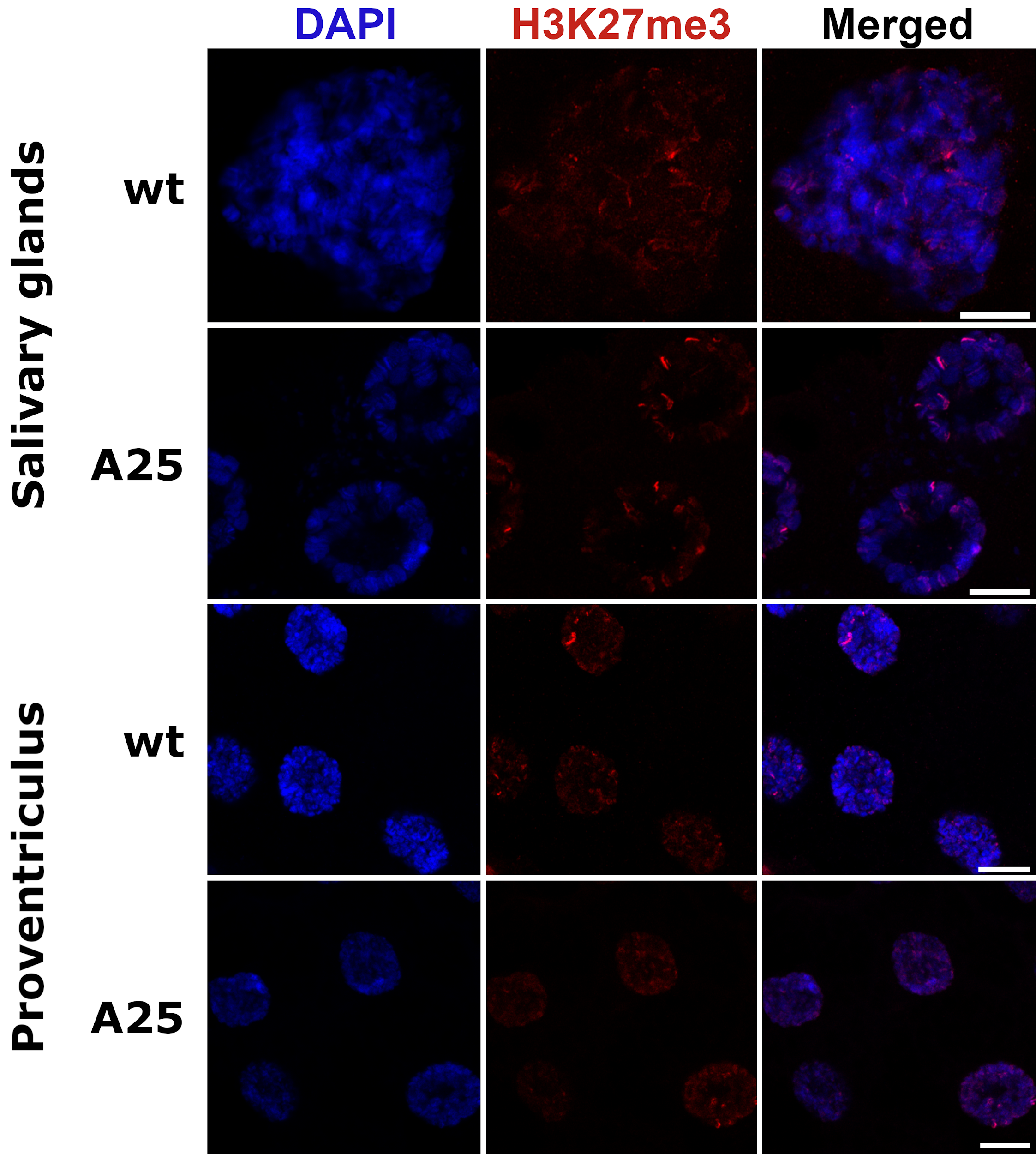


**Figure S8.** Localization of H3K27me3 in salivary gland and proventriculus nuclei from wild-type (top) and *Lam^A25^* mutant (bottom) *D. melanogaster* larvae. Chromatin (blue) is stained by DAPI. H3K27me3 (red) is stained by the specific antibody ab6002. Scale bar = 10 µm.
